# Review on Propolis Applications in Food Preservation and Active Packaging

**DOI:** 10.3390/plants12081654

**Published:** 2023-04-14

**Authors:** Narimane Segueni, Naima Boutaghane, Syeda Tasmia Asma, Nuri Tas, Ulas Acaroz, Damla Arslan-Acaroz, Syed Rizwan Ali Shah, Hoda A. Abdellatieff, Salah Akkal, Rocío Peñalver, Gema Nieto

**Affiliations:** 1 Laboratory of Natural Product and Organic Synthesis, Department of Chemistry, Faculty of Science, Campus Chaabat Ersas, University Mentouri–Constantine 1, Constantine 25000, Algeria; 2 Faculty of Medicine, University Salah Boubnider Constantine 3, Constantine 25000, Algeria; 3 Laboratoire d’Obtention des Subtances Thérapeutiques (LOST), Département de Chimie, Campus Chaabet-Ersas, Université des Frères Mentouri-Constantine 1, Constantine 25000, Algeria; 4 Department of Food Hygiene and Technology, Faculty of Veterinary Medicine, Afyon Kocatepe University, Afyonkarahisar 03200, Turkey; 5 ACR Bio Food and Biochemistry Research and Development, Afyonkarahisar 03200, Turkey; 6 Department of Food Hygiene and Technology, Faculty of Veterinary Medicine, Kyrgyz-Turkish Manas University, Bishkek KG-720038, Kyrgyzstan; 7 Department of Biochemistry, Faculty of Veterinary Medicine, Afyon Kocatepe University, Afyonkarahisar 03200, Turkey; 8 Department of Biochemistry, Faculty of Veterinary Medicine, Kyrgyz-Turkish Manas University, Bishkek KG-720038, Kyrgyzstan; 9 Department of Animal Nutrition and Nutritional Diseases, Faculty of Veterinary Medicine, Afyon Kocatepe University, Afyonkarahisar 03200, Turkey; 10 Department of Pathology, Faculty of Veterinary Medicine, Damanhour University, El-Beheira, Damanhour 22514, Egypt; 11 Unit of Recherche Valorisation of Natural Resources, Bioactive Molecules and Analyses Physicochemical and Biological (VARENBIOMOL), Department of Chemistry, Faculty of Science, University Mentouri-Constantine 1, Constantine 25000, Algeria; 12 Department of Food Technology, Food Science and Nutrition, Faculty of Veterinary Sciences, Regional Campus of International Excellence “Campus Mare Nostrum”, University of Murcia, Espinardo, 30071 Murcia, Spain

**Keywords:** propolis, antimicrobial, antioxidant, food preservative, packaging, sensory properties

## Abstract

Propolis is a natural hive product collected by honeybees from different plants and trees. The collected resins are then mixed with bee wax and secretions. Propolis has a long history of use in traditional and alternative medicine. Propolis possesses recognized antimicrobial and antioxidant properties. Both properties are characteristics of food preservatives. Moreover, most propolis components, in particular flavonoids and phenolic acids, are natural constituents of food. Several studies suggest that propolis could find use as a natural food preservative. This review is focused on the potential application of propolis in the antimicrobial and antioxidant preservation of food and its possible application as new, safe, natural, and multifunctional material in food packaging. In addition, the possible influence of propolis and its used extracts on the sensory properties of food is also discussed.

## 1. Introduction

Propolis is a well-known bee product largely investigated and studied for its beneficial effects. Propolis is a resinous material collected by honeybees from different parts of plants and trees, such as stems, leaves, and flowers. The used plants and trees are extremely variable and depend on the beehive environment and the surrounding flora [1,2]. Propolis of the temperate zone, the most studied and identified propolis all over the world, is collected from various trees, in particular exudates of poplar (*Populus* spp), eucalyptus, birch (*Betula alba* L.), alder (*Alnus glotinosa Medik*), and conifers [3,4]. Honeybees mix the collected plant and tree exudates with saliva. Consequently, during this procedure, bee wax will be added to the partially digested exudates [5].

The waxy nature of propolis is of great importance to the colony and is responsible for its use as a multifunctional building material. Propolis is used to seal pores, block cracks [6,7], cover the inner walls of the hive, repair combs, maintain the internal environment aseptic [8,9], and more easily defend the nest entrance [10]. In addition, propolis is used as a chemical weapon to protect the colony from diseases. Propolis is also used to embalm invaders to avoid putrefaction and to keep the internal environment aseptic [10,11,12,13,14].

Propolis’ chemical composition is very complex. Climatic characteristics, phytogeographic conditions, honeybee species [15,16], environmental conditions, and harvest season [17] are the main parameters that provide the diversity of propolis. Research on the chemical composition of propolis results in the separation, isolation, and identification of its chemical constituents. About 850 compounds have been reported until 2018 [18]. The significant component classes characteristic of the temperate zone propolis are phenolics (flavonoids and phenolic acids and their esters), terpenoids, alcohols, aromatic aldehydes, fatty acids, stilbenes, steroids, and lignans [13,15]. Tropical propolis, another type of propolis, differs greatly from the temperate type. The main classes reported from the tropical type are isoflavonoids, terpenes, organic acids and their prenylated derivatives, alcohols, and prenylated benzophenones [4,11]. Because of propolis chemical diversity, its organoleptic properties (aromatic smell, color, physical aspect) are also variable [4,19,20]. 

Propolis’s pharmacological properties have attracted scientific attention, and many reports have been published [10,13,20]. Propolis was reported to possess antimicrobial [21,22,23,24], antitumor [25,26,27], immunomodulatory [28,29], antihypertensive [30], anti-diabetic, and healing properties [31], etc. In recent last years, propolis found numerous uses as a component in cosmetic and pharmaceutical products. Propolis is used in anti-acne creams, body and facial creams, lotions, ointments and many other products mainly used as hygienic products for body, face, or oral hygiene [3,19]. Propolis has been used as a remedy and in supplementary medicine since ancient times. It is one of the most popular and used bee products. However, its use as a medical agent is very challenging due to the lack of standardization [19].

Food preservatives can originate from a natural or synthetic source. Their incorporation in food prevents spoilage and undesirable changes. However, chemical preservatives are considered the main cause of carcinogenicity, teratogenicity, and toxicity [32,33]. In addition, the tendency of consumers to prefer natural preservatives has greatly influenced the food industry [34]. Natural products such as essential oils, plant extracts [35,36], and bee products have been highlighted for use in foods as natural preservatives [37,38,39].

Propolis possesses recognized antimicrobial and antioxidant properties. Both properties are characteristic of food preservatives. Moreover, most propolis components, in particular flavonoids and phenolic acids, are natural constituents of food. Several studies suggest that propolis could find use as a natural food preservative [37,38,39]. This review is focused on the potential application of propolis in the antimicrobial and antioxidant preservation of food and its possible application as new, safe, natural, and multifunctional material in food packaging. In addition, the possible influence of propolis and its used extracts on the sensory properties of food is also discussed.

## 2. Actual Application of Propolis

The antimicrobial and antioxidant properties of propolis were extensively studied. In addition, propolis is recognized as safe (GRAS) [40] and can be considered as an alternative to chemical preservatives [33,41]. Before its use in food products, propolis was in most cases extracted using different solvents such as ethanol, water, and glycerol. Each solvent offers advantages and disadvantages [42]. Ethanol extracts (PEE) are reported to be the richest extracts in bioactive components [43]. While water extracts (WPE) are the lowest extract on phenolic content [44]. In contrast, the two extracts exhibited a strong flavor and aroma [44,45]. Several studies were performed on the potential applications of propolis in food preservation, in particular as a natural and safe antimicrobial and antioxidant preservative. Propolis was tested in different food formulations, including fruits, vegetables, juices, meat, seafood, oils, etc. [46,47,48,49,50,51,52,53].

### 2.1. Fruits and Vegetables

Several studies were focused on the effect of propolis extract on fruits and vegetables’ quality and storage. Propolis can be applied to the food surface or incorporated into the food formulation [42]. Propolis adjunction to food can provide health benefits. In addition, it was reported to prevent lipid oxidation and to improve shelf life [38,54]. The tested vegetables or fruits, propolis origin, and the used extracts are reported in Table 1.

In fresh whole-head and ready-to-eat lettuces (*Lactuca sativa.* L.), propolis was found to be efficient in reducing the microbial contamination [55]. Propolis used as an agricultural antimicrobial agent of cucumber crops and soybean reduced the disease severity of the two tested crops [56]. Similar results were reported for tomato fruits [57].

Propolis can be used alone or in combination with plant or essential oil. Propolis combined to gallic acid and tea essential oils was tested as a natural preservative for fresh celery, leek, and pumpkin. Both tea and propolis were found to control and reduce *E. coli* (endogenous and inoculated strains). In addition, propolis was reported to extend the shelf life and to improve the visual quality [58]. Similar results were reported for fresh-cut mixed vegetables for soup [48]. Another interesting combination is the use of propolis, chitosan, and thyme essential oil. The combination was effective on the “Vesuviono” tomato. However, chitosan was the most effective [59]. Propolis reduced the rate of weight loss and maintained fruit firmness (tomatoes) when applied as bio-coating [60]. In addition, propolis used as a coating agent can extend the shelf life of cherry tomatoes and delay the deterioration of their sensory traits [61]. In a recent investigation, pullulan film associated with ethanol extract of propolis was found to inhibit the growth of microorganisms in cherry tomatoes. The overall quality of tomatoes coated with propolis was reported to be very high, which could result in high consumer acceptance [62].

Propolis was found to decrease the population of spoilage microorganisms in potatoes [63] and to be effective in controlling potato soft rot. [64]. Propolis, cinnamon oil associated with gum Arabic were found to be an effective bio-fungicide for postharvest chili [65]. Different propolis extracts (hexane, dichloromethane, and ethanol) were reported to be efficient on molds and bacterial colonization inhibition during rice storage [66].

Postharvest storage of fruits is limited because of fruit spoilage, which can be caused by bacterial or fungal growth [42]. In addition, many other factors can be pointed out. For sweet cherries as well as many other fruits, fungal decay, weight and acidity losses, softening, stem browning, and color changes are of great importance [67].

The ability of propolis extracted in water and ethanol (70%) to prevent fungal decay in cherries stored at 0 °C for 4 weeks was tested alone and in combination. Cherries were dipped in different treatments. Ethanol treatment was the most active. In contrast, it affected stem cherries’ color and sensory quality [67]. In a recent study, propolis was stated to effectively reduce weight loss and respiration of sweet cherry and to delay soluble solids, titratable acid, and sweet cherry hardness [68]. Propolis was also active in the in vitro and in vivo inhibition of green and blue molds in *Citrus* (*Pinicillium. Digitatum* and *Pinicillium. italicum*). Moreover, no negative influence on the quality of *Citrus* fruit was reported [69]. Similar results were reported for propolis combined with *S. vanrijiae* [70]. Postharvest storage of several other *Citrus* species was also improved by propolis, such as mandarins [69] and oranges [71,72,73,74].

Propolis was effective in preservation of different fruits. Propolis protected Star Ruby grapefruit from fungal decay [75] and improved cold storage of table grape, cv. Muscatel. [76]. In addition, propolis decreased conidial germination and mycelial growth in dragon fruit [77] and control toxin production and penicillium decay [78]. Propolis was also reported to exhibit a significant antifungal activity on the growth of several strains—in particular, *P. expansum*, *Fusarium* sp, *A. alternat*, and *A. niger* isolated from apple fruit [79]. Propolis also reduced the development of anthracnose on mango fruit (variety Kent) [80] and caused a higher pulp firmness and a lower soluble solid in ‘Hindi-Besennara’ mango [81]. 

Banana is one of the most consumed fruits around the world. Different propolis types were tested in the post-harvested conservation of bananas as a coating and dipping solution. Propolis treatment caused a lower fresh weight loss in banana cv. Prata (*Mica sapientum* L.) [82,83]. In a more recent research, the crown of banana fruit was coated with 50% propolis and paraffin alone and mixed together to control crown diseases and compared to 250 ppm prochloraz and non-treated fruit. Simulation for transportation, storage, and the retail market was performed. The coating using propolis combined to paraffin was the most effective treatment showing the same results as using prochloraz [84].

In the last year, many investigations were performed on propolis and its application as a bio preservative of fruits. Pullulan, an exopolysaccharide produced by *Aureobasidium pullulans* (fungus) and one of the most used natural coatings substances, was mixed with 5% and 10% propolis ethanol extract (PEE) and tested on the shelf life of blueberry (*Vaccinium corymbosum*) fruit. The coating mixture reduced the number of molds and bacteria, delayed blueberry ripening, and decreased weight loss [85]. In the same context, edible coating with chitosan nanoparticles and 10, 20, and 30% propolis were stated to act positively on the antioxidant activity and the shelf life of strawberries. In addition, the coating did not affect the sensory characteristics of strawberries [86]. Chitosan and propolis were also effective in the preservation of quality and sensory in fig fruit. In addition, the coating substance inhibited the growth of *Aspergillus flavus* by 20–30% under laboratory and semi-commercial conditions. The coated fig fruit produced lower aflatoxin [87]. Propolis was reported to inhibit up to 90% of *S. vesicarium* mycelial growth in vitro. In artificially wounded and inoculated Rocha pear (*Pyrus communis* L. cv Rocha), propolis decreased the disease incidence and the diameter of lesions by 25% and up to 57%, respectively [88].

**Table 1:** Application of propolis extracts in fruits and vegetables.

Foods	Geographical Origin	Used Extracts	References
Vegetables			
Lettuce	Portugal (Bragança, 41°48′ N; 06°45′ W)	2% Metanol extract	[55]
Cucumber and soybean	Brazil (Pato Branco)	PEE	[56]
Celery, leek and butternut squash	Argentine (Juricich, Mendoza)	PEE	[48,58]
Tomato, lettuce seeds, onion roots	Argentine (Tucumán, Santiago del Estero, Chaco)	PEE	[57]
Tomato	Italy	Glycolic extract	[59]
Tomato	Bandung	PEE diluted with propylene glycol	[60]
Cherry tomato	China	PEE	[61]
Cherry tomato	Poland Toruń County (53.03′ N, 18.62′ E)	PEE + pullulan	[62]
Mashed potatoes	Jordan (naour region)	Nd	[63]
Potatoes	Argentine (semi-arid regions of Salta, Santiago del Estero and Tucumán)	PEE	[64]
Chili	Brazil	PEE	[65]
Rice	Brazil (Minas Gerais)	Ethanol, methylene chloride and hexane extract	[66]
Fruits			
Sweet cherry	Turkey (Hatay province (Eastern Mediterranean region of Turkey))	WPE and PEE	[67]
Sweet cherry	China	Propolis fresh liquid	[68]
Citrus	China (Baoding County, Hebei Province)	PEAE (propolis ethyl acetate extract)	[69]
Mandarins	China (Baoding County, Hebei Province)	PEAE	[69]
Orange	Egypt	PEE	[71]
Orange	Iraq (Baghdad)	Hydro-alcohol extract	[72]
Orange	Brazil (southern state of Paraná)	PEE	[73]
Citrus reticulata Blanco	Indonesia	PEE	[74]
Star Ruby grapefruit	Turkey (Hatay province)	PEE	[75]
Table grape, cv. Muscatel	Spain (Bonamel Organic S.L. (Alquería de Aznar, Spain)).	PEE	[76]
Dragon fruit	China	PEE	[77]
Apple	Iraq (Baghdad)	PEE	[78]
Apple	Egypt	PEE	[79]
Mango	Brazil	PEE	[80]
Mango	Saudi Arabia	PEE	[81]
Banana	Brazil (Bambuí, Minas Gerais)	Hydro-alcohol extract and aqueous extract	[82]
Banana	Saudi Arabia (Hada Al-Sham 21°48′3′′ N, 39°43′25′′ E)	PEE	[83]
Banana	Thailand (Ratchaburi province)	PEE	[84]
Blueberry	Poland (Torun County)	PEE	[85]
Strawberry	Mexico	-	[86]
Fig	Mexico	-	[87]
Pear	Portugal (central coast and the northern region)	PEE	[88]

### 2.2. Beverages

Pasteurization is one of the most commonly used techniques applied in food industries to improve the preservation and stability of fruit juices. As an advantage, pasteurization will prolong the duration of storage of fruit juices. However, pasteurization, in particular thermal, may accelerate the degradation of some functional compounds present in fruit juices, such as lycopene [89,90]. Chang et al. [90] used a multivariate statistical approach to analyze the previous experimental data and to demonstrate whether propolis could be used as a bio preservative in fruit juices. As a result, propolis could be used as a bio-pasteurization agent. Propolis, with its dual antioxidant and antibacterial effect, will be an interesting alternative to conventionally used techniques [90]. In this effect, propolis was effective and exhibited antifungal activity on three isolates of *Penicillium* spp. and one isolate of *Zygomycetes* spp. in four unpasteurized fruit juices (mandarin, grape, orange, and apple) [91]. Propolis was also reported to be as effective as sodium benzoate and potassium sorbate on yeast and mold inhibition [54,92,93], Propolis was also effective in apple juice [52,94], freshly squeezed juice [95], and red fruit juice [96]. Propolis was also reported to increase the phenolic content of strawberry juice. A positive effect on the color value was also observed. Sensory data determined that the effect of the addition of PE (propolis extract) on the taste, smell, color, sourness, and general acceptability of strawberry juice samples was statistically significant [97]. 

**Figure 1:**
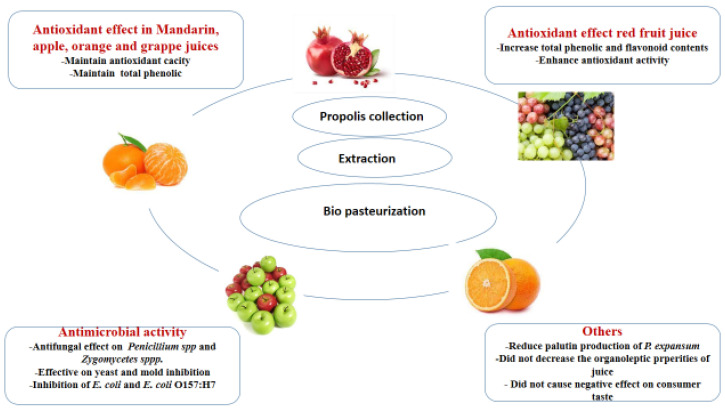
Effect of propolis on fruit juice.

The effect of propolis on fruit juice is represented in Figure 1.

The studies we described in the present review indicated that propolis has been widely tested in the preservation of fruits, vegetables, and fruit juices. The results demonstrated the effectiveness of propolis and its positive effect on the preservation of the deterioration caused by oxidation process or food spoilage. Propolis also seems to increase the levels of flavonoids and polyphenols, resulting in an increase in the antioxidant effect which will in turn slow down the oxidation of the tested products. However, opinions on its effect on the physical and sensory aspect remain mixed. Propolis concentration or percentage, used method for preservation, and duration will be the main factors to consider.

### 2.3. Dairy

Dairy products are the most consumed food products due to their high nutritive values. Dairy food items are highly perishable foods with a very short shelf life. Therefore, quick and optimized processing and storage conditions are necessarily required after milking. Today, many studies are carried out to extend the shelf life of dairy products. The substances used in these studies should be natural, accessible, economical, and in a structure that does not change the physical, sensory, and chemical properties of the product used. Propolis is seen as a high-potential option that meets this requirement [98,99].

Shaban and Galal [100] investigated the fortification and preservative ability of PE in raw and pasteurized milk. Milk’s organoleptic properties were not affected. Moreover, Propolis increased flavonoids, phenolics, and antioxidant properties. Propolis addition to milk did not increase milk acidity compared to the control group. In addition, propolis reduced bacterial counts, and no mold and yeast were detected until the 14th day. The reported results are very promising and indicate that propolis had a positive effect on the shelf life of raw and pasteurized milk. Therefore, propolis is recommended and can be used as a natural preservative (Table 2). Propolis at 5% inhibited *Staphylococcus aureus*, *Bacillus cereus*, *Listeria monocytogenes*, and *Pseudomonas fluorescence* on pasteurized milk, skimmed milk, and cheese (prepared with goat’s and cow’s milk). The outcomes of the investigation revealed that PE could significantly be applied to ready-to-eat milk products to avoid the growth of *L. monocytogenes* [101]. The co-administration of PE with glycerol exhibited a potent inhibitory activity against *L. monocytogenes* in chocolate-flavored milk. Moreover, the chocolate-flavored milk administered with glycerol and deodorized PE gained a considerable consumer acceptance rate without any negative comments or complaints. As a conclusion, propolis may exhibit an auspicious role in preserving dairy products [102]. El-Deeb [103] also suggested that propolis can effectively preserve dairy products such as milk and yogurt with improved shelf life. Gunes-Bayir et al. [104] produced yogurts with starter cultures containing different concentrations of cinnamon and propolis. The chemical analysis showed that the fat ratio decreased in propolis and/or cinnamon-treated groups. While only propolis-applied yogurt had a lower pH value, yogurts with the highest percentage and only cinnamon had the highest pH value. In the titratable acidity data, there was an increase in the groups to which only propolis was applied. In contrast, the titratable acidity of yogurts decreased in the groups in which propolis and cinnamon were applied together. In addition, a significant decrease in the colony count of *Lactobacillus acidophilus* was measured in all groups. Propolis incorporation in yogurts increased the number of *Streptococcus thermophilus* colonies. Regarding sensory properties, Chon et Al. [105] reported that the taste values of market milk, kefir, and yogurt evaluated after various propolis addition in sensory properties were similar to the control group or lower. Bilici et al. [106] reported similar results. Propolis was used to produce yogurt with improved functional properties. No change was observed in taste, smell, or color (Table 3). Propolis tested on fruit yogurts affected the titratable acidity and increased total phenolic content and DPPH inhibition in a dose-dependent manner [107].

Tumbarski et al. [108] aimed to extend the storage period of cheddar cheese with propolis incorporated in 1% carboxymethyl cellulose (CMC) edible films at various concentrations. All tested groups were kept for 56 days at 4 °C. The titratable acidity values were reduced. The lactic acid in cheese did not affect the bacterial count and did not change the increased yeasts count. However, fungal development on the surface of the cheese was inhibited, and no indications of deterioration were noticed during 56 days of storage. Considering these data, propolis application can be considered a dose-dependent effective tool in inhibiting fungal spoilage of cheddar cheese. In another study [109], the mesophilic aerobic microorganisms on the cheese’s (Gorgonzola) surface were examined. Staphylococcus was the most common bacteria. Propolis at 5.0% did not show any change in taste and odor and can be applied safely. The PE inhibited the key yeasts and bacteria without disturbing the cheese’s sensory properties. The present data showed that propolis has the potential and feasibility of application in Gorgonzola-type cheeses [109].

Propolis was found to completely inhibit the growth of *S. thermophilus*, *B. bifidium*, and *L. bulgaricus* in kareish cheese in the groups applied up to 600 and 1000 mg. Moreover, a negligible difference in the moisture content of the cheese after applying PE associated with a slight decrease in the amount of moisture and total protein was observed. According to the obtained results, propolis can be an ideal natural preservative [110].

Mehmetoğlu et al. [111] reported that propolis-added ice creams during storage did not significantly affect the physical properties of ice cream mixes. In contrast, propolis affected all sensory properties except the structure and consistency. The outcomes revealed that the addition of propolis negatively affected ice cream’s physical and sensory properties.

### 2.4. Meat

**Figure 2:**
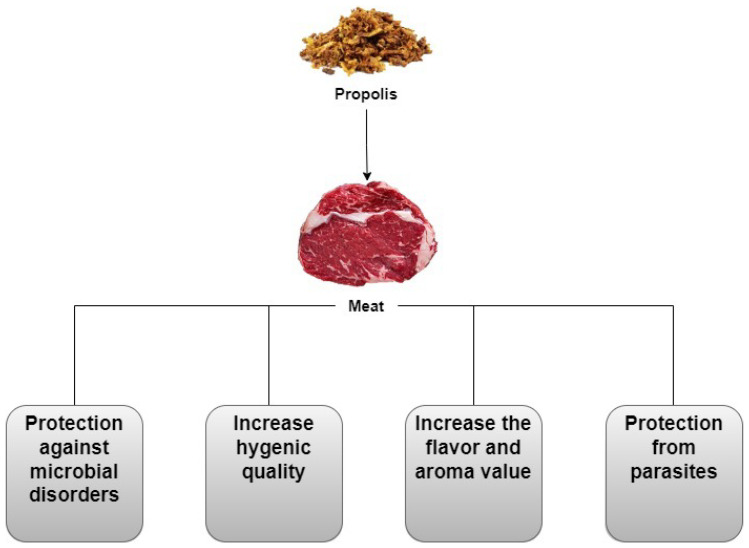
Effect of propolis on meat.

Meat and numerous meat products are crucial sources of several nutrients such as proteins, amino acids, minerals, and different vitamins. Meat products are an essential part of the human diet due to their nutritional value. Several studies have reported propolis antimicrobial and antioxidant potential to improve the shelf life of meat and different meat products without causing adverse effects (Table 2). The effects of propolis on meat products are shown in Figure 2.

Safaei and Roosta [112] prepared active polylactic acid (PLA) films containing PE to increase the shelf life of sausages. The tested film showed the highest inhibition zone against *S. aureus* compared to *P. aeruginosa*, suggesting that PE can provide potential antimicrobial activity for long-term storage. Lipid oxidation is among the most important deteriorations in meat. Lipid oxidation produces bad odors and tastes and negative changes in the texture, color, and quality of the product. Therefore, controlling lipid oxidation with the active packaging method containing antioxidant substances will positively contribute. The total phenolic content in the processed sausage samples was increased when they were packaged with PE-containing films. PE can effectively be used to prepare PLA-based active films to enhance the shelf life of meat sausages [112].

A chitosan (Ch)-based coating containing a mixture of *Zataria multiflora* essential oil (EO) and propolis (PE) was prepared and tested for its ability to prevent spoilage and enhance the shelf life of breast meat chicken [113]. The effect of packaging material was evaluated on the poultry meat’s microbial, chemical, and sensory properties. The coating mixture reduced the presence of psychrotrophic bacteria on all days of storage. Chitosan can cause a decrease in the pH of chicken meat. Propolis addition did not cause any change in color parameters [113]. Vargas-Sánchez et al. [50] aimed to evaluate the lipid oxidation and the antimicrobial effect of non-commercial and commercially available PE on beef patties stored in refrigerators. Raw beef patties were kept at 2 °C for a duration of 8 days with polyvinyl chloride (PVC) wrapping. The microbial count, lipid oxidation, color, and pH parameters were determined. The non-commercial PE showed the most effective results for all the tested parameters. In addition, lipid oxidation was inhibited, and microbial counts were reduced. No significant changes were observed in the pH of propolis extract treated groups. Moreover, the addition of PE to patties maintained the fresh red color for 8 days compared to the control. These results demonstrated that PE could be used as a natural antimicrobial and antioxidant agent for prolonging beef shelf life.

Another study [114] evaluated the effectiveness of turmeric and propolis powders as natural preservatives with different concentrations (1.5–2.5%) in packaged minced meat. Both propolis and turmeric aqueous extract inhibited *Fusarium oxysporum*, *L. monocytogenes*, *P. aeroginosa*, and *Saccharomyces cerevisiae.* Propolis was less effective on Gram-negative bacteria compared to Gram-positive bacteria. An increase in pH values was observed during storage by applying propolis and turmeric at various rates, and the highest increase was obtained in the control groups [114]. An enriched chitosan (Ch) film combined with cellulose nanoparticle (CNP) and PE was developed and tested on chemical, microbial (*Pseudomonas* spp. and *Enterobacteriaceae*), and sensory properties of minced beef. Applying Ch prolonged the shelf life of minced meat up to 6 days when it was kept in the refrigerator. The prepared films with different compositions, such as 2% PE, 2% CNP with 2% PE, and 1% CNP with 2% PE, exhibited a significant delay in protein and lipid oxidation and microbial count in minced meat [115]. 

Similar results were reported by Gedikoğlu [116] for raw beef patties. Propolis was most active against *S. epidermidis*, followed by *E. faecalis* and *L. monocytogenes*. The PE decreased the total number of mesophiles. Color is an excellent marker of the freshness of meat products and is a critical factor for consumers’ choices. The microbiological and oxidative changes affect the meat color, leading to discoloration through metmyoglobin formation. In this analysis, all color values were reduced in the post-test time for metmyoglobin formation. In addition, the redness value was reduced with PE addition.

Mafra et al. [117] investigated propolis’s physicochemical, microbiological, and sensory properties on tilapia salami. No change was observed in the color, moisture, lipid, and weight values. Propolis application does not cause any change in the acceptance of consumers and it has been proven that there is no change in sensory properties. Özkır [118] investigated the use of propolis in the production of meatballs. Propolis did not cause any change in the Aw values of all tested meatball samples. Propolis addition caused a significant decrease in the thiobarbituric acid (TBA) values and regulated the oxidation stability during storage. The analysis of total phenolic content revealed that the addition of propolis significantly increased the total phenolic content and antioxidant capacity of the meatballs. The sensory analysis indicated that, at high propolis concentration, a sharp taste and pronounced smell of propolis was observed. Consequently, satisfaction with the sensory analysis decreased.

Propolis was added to Italian salami and kept for 90 days. Physical properties analysis showed that the inclusion of propolis did not affect the drying process of Italian-style salami, and no effect was observed on the lightness of the salami during its shelf life. The addition of propolis did not change the acceptability of the product. Regarding sensory properties, the judges rated the flavors as smooth and pleasant. Still, some judges detected the smell of propolis in the salami, which led to less acceptance of the salami. After the 90-day storage period, the majority of consumers did not comment on the presence of a sour flavor in the products. This confirmed that the salami remained stable in terms of lipid oxidation. Some consumers have noted that salami has an unpleasant, strong taste residue. However, they could not state that this aroma comes directly from propolis [119]. The organoleptic and physicochemical changes of propolis were investigated on sausages. The pH decreased until the 8th day, but after the 16th day, it began to increase due to enzymatic denaturation during storage. The volatile nitrogen bases (TVB-N) were also increased. A correlation was observed between the pH and TVB-N increase on the 16th day. In addition, TBA values showed that lipid oxidation increased up to the 24th day. Sensory investigations revealed that the taste and smell of the cooked product were good. Most of the scores were within the acceptable range. There was no significant difference in sensory evaluation between the control and propolis-treated groups. Consumers accepted the sensory properties of propolis sausages. However, they suggested improving the color. The physical properties analysis also suggested that propolis could be used as an alternative for the natural preservation of meat products [120].

### 2.5. Chicken

Chicken meat is the most economical and widely consumed meat source as compared to mutton or beef. Chicken meat containing proteins, vitamins (such as pantothenic acid, thiamin pyridoxine, and thiamine), fats, and minerals (Cu, Zn, Fe, etc.) is highly nutritive for the human body. The presence of high moisture levels and proteins in chicken meat facilitates microbial growth, reducing the quality and shelf life of chicken meat. Several preservatives are in use to avoid spoilage of chicken meat [113]. Propolis is emergingly being applied in the food industry to preserve food products with enhanced shelf life (Table 2). 

Jonaidi Jafari et al. [121] reported that Ch and PE could be used together to extend the shelf life of chicken fillets. Propolis extract was sprayed on chicken fillets or combined with chitosan. If PE is sprayed, the concentration decreases over time due to its high volatility, resulting in a less antimicrobial effect. However, co-administration of PE and Ch helps the extract retain its antimicrobial properties for a long time. Color and texture changes in propolis-treated groups were not significantly different compared to the control group. However, the acceptance of smell and taste decreased. Higher amounts of propolis may reduce some sensory properties of fillets. Despite of some adverse effects on the sensory values of the groups, Ch combined with PE could be used to extend the shelf life of the chicken fillets.

Another investigation [122] evaluated the effects of propolis on immune response and meat quality in broilers infected with *Escherichia coli.* The number of Coliforms, Staphylococcus, and Psychrotrophic bacteria decreased in the chest muscle of the PE-treated group. Considering the sensory characteristics, the PE-treated group revealed the highest score in general acceptability. The PE-treated group revealed an increased body weight, decreased mortality, decreased rate of *E. coli* re-isolation from internal organs, and accelerated infection recovery. Pochop et al. [123] tested the effect of PE in chicken feeds against *Salmonella* spp. colonization on the gastrointestinal tract. The results showed that all experimental groups treated with PE could inhibit and eliminate *Salmonella* spp., suggesting that propolis positively impacts *Salmonella* spp. in the gastrointestinal tract. This study is not only beneficial to prevent humans from being infected with *Salmonella* but could also be interesting for food manufacturing companies and may be explored in the extension of the shelf life of food products and saving the cost of storing food products while awaiting pathogen test results. 

Kročko et al. [124] studied antibiotic resistance changes in chickens after exposure to propolis and bee pollen. In general, *Enterococci* are found in the normal microbiota of the gastrointestinal tract of chickens but are not considered safe. Intermediate resistance to erythromycin disappeared in most *E. faecalis* isolates of broiler chickens fed propolis in their diet. Likewise, ampicillin and gentamicin resistance decreased after bee pollen application. However, it was noted that the sensitivity of *E. faecalis* to vancomycin and teicoplanin did not show any change in propolis and bee pollen compared to the control. The results showed that antibiotics could be used in association with propolis and bee pollen in broiler chickens’ diets. Mahdavi-Roshan et al. [125] reported that the use of PE (up to 8%) did not negatively affect the color and general acceptance of chicken breast. In addition, positive increases were observed in the texture values. The significant results of this investigation in the era of the poultry industry have opened the door to naturally effective preservatives. Solving poor quality and shelf life problems using PE-applied products will benefit public health and the economy.

### 2.6. Fish

The aquaculture is susceptible to develop different bacterial infections, particularly when nurtured in dense conditions. The occurrence of infections in aquaculture is responsible for causing a reduction in the production rate and an escalation in mortality, resulting in economic shortfalls [126]. Antibiotics were used to treat the bacterial infections in aquaculture. The escalated use of antibiotics in fish farms induced an antibiotic resistance in humans and animals. For example, the emergence of resistance was observed for *Aeromonas salmonicida*, *A. hydrophila*, *Yersinia ruckeri*, *Vibrio salmonicida*, *V. anguillarum*, *Edwardsiella icttaluri*, *E. tarda*, and *Pasteuralla piscida* [127]. Some fish microbes are highly pathogenic to cause diseases in humans via food-borne or zoonotic routes. Therefore, it is critically required to develop potential antimicrobials to treat microbial illness in fish. As natural products are a focus of researchers nowadays, propolis has been found to possess significant antimicrobial as well as antioxidant activity. Numerous studies have reported the significance of propolis in terms of providing preservative effects in food (Table 2).

Abd-El-Rhman [128] investigated the efficacy of PEE against *Aeromonas hydrophila*. Crude propolis (1%) was added to the feed already containing 30% crude protein. About 225 species of *Oreochromis niloticus* fish were used. PEE increased the growth, resistance, and immunity of *Oreochromis niloticus* against *Aeromonas hydrophila* more than crude propolis. Tukmechi, Ownagh et al. [129] investigated the effect of PEE against three pathogenic bacteria that are common in fish (*Streptococcus iniae*, *Aeromonas hydrophila,* and *Yersinia ruckeri*). Enrofloxacin and gentamicin were used as references. The PEE was found to have a significant antimicrobial activity against *Streptococcus iniae* and was able to inhibit the growth of *Aeromonas hydrophila* and *Yersinia ruckeri*. Based on the potential antibacterial properties, propolis can be used in aquaculture, and more beneficial applications can be explored.

The PE (2, 8, 16%) was used to prepare gelatin films [130] and evaluated for the preservation of trout fillets. At the end of storage, all PE-treated groups showed a significant reduction in the growth of mesophilic and psychrophilic bacteria. In addition, mold and yeast counts were also reduced. Based on the obtained results, it was concluded that the 16% concentration of PE showed the highest antimicrobial activity against all tested microorganisms. The sensory were affected by the increase in time. It can be suggested that PE-containing films can be used to maintain and enhance the quality of trout fish by controlling microbial deterioration and improving the sensory properties.

Kuley et al. [131] prepared a microencapsulation containing PE and *Lactobacillus plantarum* to investigate the antibacterial potential against fish pathogenic bacteria. Different microencapsulations were prepared with cell-free supernatant (CFS) of *L. plantarum* and three different PE. Antimicrobial activity of pure CFS *L. plantarum*, microencapsulated CFS *L. plantarum* (ECFS), CFS *L. plantarum* microencapsulated with water-soluble propolis extract, and CFS *L. plantarum* microencapsulated with PE was determined against *Pseudomonas luteola*, *Proteus mirabilis*, *Photobacterium damselae,* and *E. faecalis* using agar well diffusion and broth microdilution methods. Inhibitory results were shown for *P. damselae* with the most effective antimicrobial activity. Pure *L. plantarum* showed the highest inhibitory activity for *P. damselae* and the lowest for *P. mirabilis*. Microencapsulated *L. plantarum* showed the highest inhibitory activity for *P. damselae* and the lowest for *P. mirabilis*. APE microencapsulation showed the highest inhibitory activity for *P. luteola* and the lowest for *E. faecalis*. PEE microencapsulation showed the highest inhibitory activity for *P. damselae* and the lowest for *P. luteola*. Microencapsulated with water showed the highest inhibitory activity for *P. damselae* and the lowest inhibitory activity for *E. faecalis*. Prepared microcapsules can be added directly to food formulations due to their powder form and easy dissolution and can be applied as a new antimicrobial agent in food products.

Ebadi et al. [132] synthesized PE-containing chitosan (Ch) coatings to improve the shelf life of fish (*Nemipterus japonicus*) fillets. The fillets treated with PE-incorporated Ch coatings showed lower bacterial and significantly retard the decomposition of proteins and oxidation of lipids. The synthesized coating could maintain or strengthen the sensory characteristics ranging in acceptable limits and increase the shelf life of *Nemipterus japonicus* for about more than 10 days. Based on the obtained results, the PE-incorporated Ch coatings were found to be a potent antimicrobial and antioxidant material and can act as an effective method to maintain and improve the quality of fish fillets storage with an extended shelf life. 

**Table 2:** Impact of propolis on different food products.

**Food Product**		**Propolis Type**	**Treated Microbe**	**Origin**	**Chemical Composition**	**Reference**
Dairy	Buffaloes’ milk	PE	Total bacterial count, coliforms, molds, and yeasts	Plant Protection Department at the Faculty of Agriculture, Mansoura University	Phenolic compounds (11.18 ± 0.511 mg/g) Flavonoids (7.716 ± 0.587 mg/g) Antioxidant activity (70.44 ± 0.327%)	[103]
	Milk	PEE	*L. monocytogenes*, *S. aureus*, *B. cereus*	Val di Cecina (Tuscany, 50–450 m above sea level)	Flavonoids (2.3%)	[101]
	Yogurt	PE	*B. animalis ssp*. *lactis*, *L. delbrueckii subsp. bulgaricus*, *L. acidophilus*, *S. thermophilus*	Purchased from Sepe Natural Organic Products (Turkey)	300 mg of Brazilian propolis (3.5 mg Gallic Acid and 2.1 mg Quercetin per mL product)	[104]
	Gorgonzola type cheese	Brazilian green PEE	*S. equorum*, *S. saprophyticus*, *C. parapsilosis*, *S. cerevisiae*, *C. flavescens*	Campo das Vertentes (Latitude 21 140 S. Longitude 44 590) in the state of Minas Gerais, Brazil	ND	[109]
	Kareish Cheese	PEE	*S. thermophilus*, *B. bifidium*, *L. bulgaricus*	Plant Protection Department at the Faculty of Agriculture, Mansoura University	Phenolic Compounds (13.64 ± 0.440 mg/g), Flavonids (10.57 ± 0.605 mg/g), Antioxidant activity (67.12 ± 0.288%)	[110]
	Milk	PEE	*L. monocytogenes*	Collected in June from Megalopolis (MEG), in the region of Arcadia (central Peloponnese), Greece	Phenolic acids and derivatives (1.42 ± 0.04 mg/g), Flavones and flavonols (6.20 ± 0.32 mg/g), Flavanones and dihydroflavonols (3.56 ± 0.11 mg/g)	[37]
	Kashkaval Cheese	PE	Lactic acid bacteria, yeast, and fungi	Purchased from producers from three different regions in the Western part of Bulgaria—town of Simitli, Blagoevgrad district (41°53′ N 23°7′ E), town of Bankya, Sofia district (42°42′ N 23°8′ E), and village of Vladimir, Pernik district (42°26′ N 23°5′ E).	ND	[108]
	Pasteurized and raw milk	Crude organic propolis	Total microbial count	New Valley, Dakhla	ND	[100]
Meat	Dry meat sausage	Raw dark brown propolis	*S. aureus*, *P. aeruginosa*	Honey Mahvin Company	Phenolic content (27.08 ± 2.4 mg/g)	[112]
	Chicken breast meat	PE	*Pseudomonas* spp.	Natural propolis was gathered from four beehives.	Flavonoids (25.2%), Sesquiterpenes (9.5%), Aromatic acids (5.01%), Aliphatic hydrocarbons (4.87%), Aromatic hydrocarbons (5.80%), Fatty acids (2.24%), Alcohol (2.05%), Aldehydes (1.45%), Triterpenes (1.12%)	[113]
	Beef patties	PE	Mesophilic and Psychrotrophic	Commercial propolis samples 1 and 2 Non-commercial propolis; Pueblo de Alamos, Sonora, Mexico (29°07′129′N)	Cinnamic acid, Rutin, Myricetin, Quercetin, Chrysin, Kaempferol, Apigenin, Pinocembrin, Luteolin, Acetin.	[51]
	Minced beef	PE	*L, monocytogenes*, *P. aeruginosa*, *F. oxysporum*, *S*. *cerevisiae*	National Research Center in Cairo, Egypt	Pentacosane (0.36%), 5,5-D2-Trans-4,3-Dihydroxycylopentene (0.64%), Dodecane (1.04%), Trans-cyclohexanol,2-(methylaminomethyl) (7.30%), 4-aminocyclohepta[f]thieno[2,3-b]pyridine (66.02%), A-Neooleana-3(5), 12-diene (22.99%), 4-Methoxyamphetamine (1.66%)	[114]
	Minced beef	PEE	*S. aureus*, *B. subtilis, B. cereus*, *L. monocytogenes*, *S. typhimurium* and *E. coli O157:H7*	Purchased from an apiary located in the suburb of Kermanshah, in the west of Iran	Lindane (11.96%), caffeic acid (11.14%), 3-cyano-5, 6-dihydro-4-(methylthio)- 2-phenylbenzo[h] quinoline (10.31%), buxozine-c (9.01%), 3,4-bis (3-byano-2-methylphenyl)-2,5-dimethylfuran (8.45%), 12-azabicyclo [9.2.2] pentadeca-1(14), 11(15)-dien-13-one (8.43%), and naringenin (7.31%)	[115]
	Beef Meatballs	WPE	*S. epidermidis*, *E. faecalis*, *L. monocytogenes*	ND	Antioxidant Activity (IC_50_ 38.025 ± 0.135 µg/mL) FRAP (23.27 ± 0.26 µM of Fe + 2/g)	[116]
Chicken	Chicken breast fillet	PEE	*Pseudomonas* spp.	Natural propolis was gathered from four beehives	Flavonoids (25.2%), Sesquiterpenes (9.5%), Aromatic acids (5.01%), Aliphatic hydrocarbons (4.87%), Aromatic hydrocarbons (5.80%), Fatty acids (2.24%), Alcohol (2.05%), Aldehydes (1.45%)	[113]
	Chicken fillet	PEE	*S. aureus*	Collected from different locations of Tehran Province in May 2016	ND	[121]
	120-days old chicks	Egyptian propolis	*E. coli*	Dakahlia Governorate	ND	[120]
	One day old chickens	PE	*Salmonella* spp.	ND	ND	[123]
	Broiler chickens of hybrid combination Ross 308	Propolis and bee pollen	*E. faecalis*	ND	ND	[124]
	Chicken breast	PE	Yeasts and molds, *S. aureus*, *E. coli*	Supplied from the mountainous area west of Guilan province	Phenolic compounds (5.83 ± 0.505 g/ 100 g), Flavonoids (4.92 ± 0.562 g/mL), Antioxidant activity (15.83 ± 2.341%)	[125]
Fish	Nile tilapia (*Oreochromis niloticus*)	PEE and crude propolis	*A. hydrophila*	Collected in summer from Upper Egypt	ND	[128]
	Fish	PEE	*A. hydrophila*, *Y. ruckeri* and *S. iniae*	Adana region	Propolis antioxidant activity value (569.68 µmol trolox/g), phenolic substance content (593.31 mg GAE/g)	[130]
	Rainbow trout (*Oncorhynchus* *mykiss*)	PE	Mesophilic, psychrophilic, yeast and mold, coliform	Adana region	Propolis antioxidant activity value (569.68 µmol trolox/g), phenolic substance content (593.31 mg GAE/g)	[130]
	Fish	PE	*L. plantarum*, *E. cloacae*, *P. luteola*, *P. mirabilis*, and *P. damselea*	Propolis was produced by *Apis mellifera* in January 2020, Adana, Turkey.	ND	[131]
	Rainbow trout (*Oncorhynchus mykiss*)	PE	Mesophilic and psychrotrophic bacteria	Propolis was collected from a farm at village Kocaavsar in Balikesir, Turkey.	ND	[133]
	*Nemipterus japonicus* fillets	PEE	Mesophilic and psychrotrophic bacteria	ND	ND	[134]

**Food Product**

**Propolis Type**

**Treated Microbe**

**Origin**

**Chemical Composition**

**Reference**

## 3. Propolis and Active Packaging

**Figure 3:**
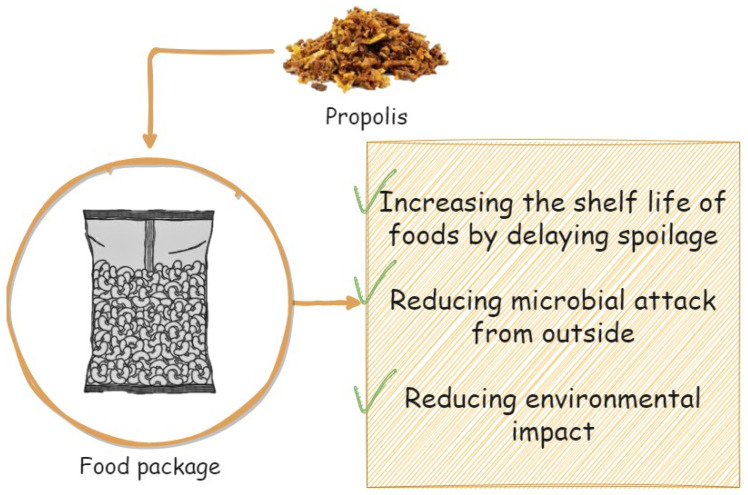
Benefits of propolis in food packaging.

Active packaging includes processes such as delaying food spoilage, preventing microbial attacks, reducing environmental efficiency, and positively affecting shelf life without compromising food quality (Figure 3). Recently, various new materials and packaging techniques have been developed to replace non-biodegradable materials for use in foods [135,136,137,138]. According to Roy and Rhim [138] plant extracts and other natural bioactive products combined with other packaging materials have better performance in preventing microbial contamination, reducing oxidation that occurs in food, and maintaining nutrition without degrading or contributing positively to sensory quality. Among these natural substances is propolis, which has recently been applied as a food supplement and alternative medicine. In recent years, propolis has gained wide acceptance in various countries’ food supplements and alternative medicine [138,139]. According to the World Health Organization (WHO), propolis can be combined with other medications without hindering conventional treatment [140].

Due to its richness in polyphenolic compounds, propolis has applications in many areas, such as active food packaging and treatment applications. Edible coatings and functional packaging films based on propolis extract have a wide range of applications in the storage of various foods such as vegetables, fruits, and meats [82,138,141,142].

Propolis extracts can be added directly to food to prevent microbial growth in food. In addition, propolis extracts are plasticizers, emulsifiers, potentiators, and a wide variety of other compounds such as chitosan [115,143], starch [144,145,146], κ-carrageenan [147], gelatin [148,149], agar [150], and biopolymers can be applied to substances. Because of this situation, the addition of propolis extracts to food packaging products or biopolymers film matrix creates opportunities for producing active packaging films that are very safe and environmentally friendly compared to petroleum-based plastic packaging films [151].

## 4. Other Applications of Propolis

Cedeño-Pinos et al. [152] tested the protective effects of PEE on jelly candies. The chemical analysis revealed that the addition of PEE increased the average values of Azinobis-3-ethylbenzothiazoline-6-sulfonic in candies made with sugar or fructans (Table 3). The addition of PEE also increased the 1, 1-Diphenyl-2-Picrilhydrazine values in sweets made with sugar or fructans. The physical properties analysis indicated that using PEE did not affect moisture, pH, CIELab color, or instrumental texture. When examining sensory traits, consumers often did not clearly distinguish between unprocessed and processed sugars. The sensory and physical properties of gummy gel were tested by adding propolis and honey. No significant changes were evaluated in tissue and Aw during the storage. Propolis inhibits the growth of microorganisms in microbial cultures and exhibits a synergistic effect when applied together with honey. It was determined that propolis concentrations higher than 30 g kg^−1^ did not show microbial growth or fungal growth in gummy jellies during 90 days of storage at 25 °C. The propolis-added gummy gel sample differed from commercial confectionery in all tested parameters for physical properties such as softer and more flexible). The gummy gel with added propolis also revealed less effort to chew. The color feature was evaluated positively by 88% of the consumers and received the most acceptance. In addition, the stickiness feature was not found sufficient by 66% of consumers, and there was no clear criticism for other features. During storage, a positive change in color and antioxidant capacity with a decrease in gloss was detected [153].

**Table 3:** Effect of propolis and its extracts on different food products’ chemical, physical, and sensory properties.

Propolis Type	Applied Product	Chemical Properties	Physically Properties	Sensory Properties	Geographical Position	Chemical Composition	Reference
Red propolis	Tilapia salami (*Oreochromis niloticus*)	The pH value of salami decreased in the first 8 days but increased after the 8th day. Aw decreased until the end of ripening in salami.	No color change was observed. No significant differences were seen in the moisture, lipids, and weight loss tests.	No off-flavor sensory change was observed.	Purchased from the Cooperativa de Apicultores de Canavieiras Coaper located in the municipality of Canavieiras (15°40′30′′ S and 38°56′50′′ W), Bahia, Brazil.	Phenols (10.37 ± 0.15 g of gallic acid/100 g of propolis) and flavonoids (3.53 ± 0.14 g of rutin/100 g of propolis)	[117]
Lactic acid-based propolis extract	Strawberry juice	The pH value was changed. Phenolic content was affected.	No adverse effects were observed at low concentrations (0.4–0.7%) but at high concentrations (1%).	A positive effect on the color was found to be directly dependent on the propolis amount.	Api10, Apipark Beekeeping Production, Türkiye	Total phenolic contents varied between 2896.19 GAE mg kg^−1^ and 512.8571 GAE mg kg^−1^.	[97]
Propolis extract	Ground beef	The pH, DPPH, and phenolic values were changed. Aw remained the same, TBA value decreased.	No changes were determined in physical properties.	The taste and odor were adversely affected.	Tekirdag Namık Kemal University, Department of Food Engineering	Total phenolic substance values (639.9 ± 4.2 mg GAE/kg)	[118]
Green propolis ethanolic dry extract	Jelly candies	There was an increase in ABTS and DPPH.	No physical changes were found.	No off-flavor sensory change was observed.	Pindamonhangaba, São Paulo, Brazil	Total polyphenols (8.71% *w*/*w*)	[152]
Raw propolis	Gummy jelly	No changes were observed in tissue and Aw.	An evident physical change was found in comparison to the control group.	The color and adhesive properties were changed.	Gualeguaychú, Entre Rios, Argentina	ND	[153]
Extrato De Própolis Milagres 30 mL (Mel Milagres Ind. Brazil)	Milk, yogurt, and kefir	Except for market milk, the pH value of yogurt and kefir did not differ between the experimental and control group.	Induced a marked difference in market milk, yogurt, and kefir color and texture.	Induced a distinct color and texture difference in market milk, yogurt, and kefir.	Purchased from Extrato De Própolis Milagres 30 ML (Mel Milagres Ind. Brazil)	ND	[105]
Ethanolic extract of propolis	Cherry tomatoes	There was no change in pH.	A better color was obtained, and post-harvest life was extended without weight loss.	The overall quality of tomatoes with propolis was measured as very high.	Purchased from an apiary located in Toruń County (53.03 m an apiary located in To	Total phenolic acids (20,028.7 mg L^−1^), Total flavan-3-ols (113.2 mg L^−1^), Total flavanone (1738.8 mg L^−1^), Total flavanonols (21,704.7 mg L^−1^), Total flavones (26,267.2 mg L^−1^), Total flavonols (13,305.4 mg L^−1^)	[62]
Raw propolis	Ice cream	No chemical changes were found.	There was little change in hardness and adhesive properties.	All sensory properties except the structure consistency were adversely affected.	Collected in August from the Apiculture Research Institute apiaries located in Dedeli Village of Ordu province in the summer season of 2018.	Total Phenolic Substance (1 g of propolis GAE mg/mL 136.19 ± 3.35)	[113]
Fanus Organic Propolis (Water Based)	Yogurt	No change in pH.	No changes were observed.	No adverse change was found in taste and odor.	ND	ND	[106]
Propolis extract	Fruit Yogurt	The pH, DPPH, and phenolic values were changed.	No changes.	No off-flavor sensory change was observed.	Collected from beekeepers in Ordu/Türkiye in September-October 2014.	Total phenolic substance (mg GAE/g 114.26 ± 2.64), Aliphatic Acids (8.17%), Aromatic Acids (4.42%), Esters (3.81%), Alcohol and terpenes (4.84%), Flavonoids (1.99%), Sugars (15.38%), Others (61.39%)	[107]
Ethanolic extract of propolis	Fresh sausages	The pH showed a fluctuating course. TVB-N and TBA increased.	A decrease in red color was observed.	Improved the taste and smell of sausages.	Obtained in Boyacá-Colombia	ND	[120]
Propolis extract	Beef Patties	The number of flora was found to be decreased.	The color was adversely affected.	No off-flavor sensory change was observed.	Obtained from an apiculture local market in Hermosillo, Sonora.	The total phenolic content in PE was 75.4 ± 0.02 and 36.8 ± 00.	[50]
Green propolis	Italian-type salami	The pH and humidity changes remained normal.	No physical changes were found.	A slight change was found in smell and taste.	ND	ND	[119]

## 5. Challenges and Limitations

There are many challenges in the use of propolis as a natural food preservative. The present review tries to summarize the potential use of propolis in food preservation. It is well established that propolis can be used as a multifunctional constituent in food and beverage products. Due to its antioxidant and antimicrobial properties, propolis can help maintain both the quality and safety of different food products.

Before its use in food products, propolis was in most cases extracted using different solvents such as ethanol, water, and glycerol. Each solvent offers advantages and disadvantages [42]. Ethanol extracts are reported to be the richest extracts in bioactive components [43], while water extracts are the lowest extract on phenolic content [44]. In contrast, the two extracts exhibited a strong flavor and aroma [44,45]. For instance, the performed studies did not conduct a chemical investigation on the qualitative and quantitative constituents to help better identify which compounds are involved in such activity and their respective concentration in the extract. This step is crucial in the standardization of propolis extract for a possible future use in food industries. Many propolis have been standardized for possible use in therapy, and some clinical trials have been carried out [154,155,156]. However, the approach to be followed should be reviewed for large-scale use in industry. The incorporated propolis extracts will be led to be heated, cooled, or even frozen as needed, which involves the study of their activity and composition throughout the food manufacturing process, starting with the addition of the extract to the final product. Another important aspect is the bioavailability and stability of propolis active compounds. Studies are in need in this field.

The limitation in the use of propolis extracts in food can be overcome by the use of encapsulation. The use of different delivery vehicles and/or forms was reported to increase propolis bioavailability and to maintain propolis antimicrobial and antioxidant properties [157]. Moreover, it can mask the unpleasant taste and aroma of propolis extracts.

## 6. Conclusions

Propolis is a natural substance harvested by honeybees from buds of different plant species, used in folk medicine since ancient times, mainly in cadavers’ conservation. It possesses potent antioxidant, antibacterial, antiviral, anti-inflammatory, and other biological activities. Constituents of propolis are variable and complex, depending on many factors such as plant species, plant origin, climate, soil type, harvesting techniques, and season. Several studies have highlighted the effectiveness of propolis in nutritional and other health-promoting properties. Due to its variable phenolic compounds as polyphenols and flavonoids, these ingredients increased the beneficial effects of propolis, and, therefore, propolis products are considered essential for different food industries.

Propolis can be applied to the food surface or incorporated into the food formulation. Its adjunction to food can provide health benefits. In addition, propolis was reported to prevent lipid oxidation and to improve the shelf life of several food products, including vegetables, fruits, and beverages. Propolis can also be applied as a safe, new, and natural preservative in meat, fish, and chicken. Moreover, the antibacterial and antioxidant properties of propolis can be used in aquaculture. However, the unpleasant taste and smell of propolis may limit its use by affecting the sensory properties such as color, odor, appearance, texture, and general taste criteria of food products.

Economically, the incorporation of propolis and its derivatives in the global food market is a worthy of consideration. Therefore, it is plausible to predict the great industrial manufacturing of propolis to be a basic product in life in the future. Hence, extensive further studies need to be achieved for the standardization of propolis and for the establishment of newly developed technologies for the incorporation of this product in nanoparticles and food systems.

## Data Availability

Not applicable.
